# Long-term results of posterior cruciate ligament tear with or without reconstruction: A nationwide, population-based cohort study

**DOI:** 10.1371/journal.pone.0205118

**Published:** 2018-10-03

**Authors:** Sheng-Hao Wang, Wu-Chien Chien, Chi-Hsiang Chung, Yung-Chih Wang, Leou-Chyr Lin, Ru-Yu Pan

**Affiliations:** 1 Department of Orthopaedics, Tri-Service General Hospital, National Defense Medical Center, Taipei, Taiwan, R.O.C.; 2 Graduate Institute of Medical Science², National Defense Medical Center, Taipei, Taiwan, R.O.C.; 3 School of Public Health, National Defense Medical Center, Taipei, Taiwan, R.O.C.; 4 Division of Infectious Diseases and Tropical Medicine, Department of Internal Medicine, Tri-Service General Hospital, National Defense Medical Center, Taipei, Taiwan, R.O.C.; Mayo Clinic Minnesota, UNITED STATES

## Abstract

**Background:**

There is increasing interest in the long-term outcomes of patients with posterior cruciate ligament (PCL) tears following conservative treatment or reconstruction. However, limited information is available regarding these results because of the relative rarity of cases and lack of long-term follow-up.

**Purpose:**

The goals of this study are to (1) compare the occurrence of secondary meniscal tears, osteoarthritis (OA) or subsequent total knee replacement (TKR) in patients with or without PCL injury, and (2) evaluate the potential protective effect of PCL reconstruction against long-term sequela in patients with PCL deficiency.

**Study design:**

Cohort study; Level of evidence, 3

**Methods:**

This retrospective cohort study evaluated the long-term results of PCL deficiency with or without reconstruction in Taiwan based on data from the National Health Insurance Research Database (NHIRD) from 2000 to 2015. The cumulative incidence rates of meniscus tear, OA and TKR were analyzed using the Kaplan-Meier estimator. Cox proportional hazards models were applied to estimate hazard ratios (HRs) and 95% confidence intervals (CIs).

**Results:**

A total of 4,169 patients diagnosed with PCL tear from 2000 to 2015 in Taiwan were included in the study. There was a higher cumulative incidence of meniscus tear (1.13%), OA (2.71%) and subsequent TKR (0.91%) among patients with a PCL tear than among patients without one (0.22%, 1.90%, 0.62%; all p < 0.05). PCL reconstruction patients had a decreased cumulative incidence of meniscus tear (0.41%), OA (2.30%) and subsequent TKR (0.48%) compared with non-reconstructed patients (2.44%, 3.46%, 1.69%; all p < 0.05). After adjusting for covariates, PCL-injured patients who underwent reconstruction within one year after PCL injury showed a significantly lower risk of subsequent sequelae than those who never underwent reconstruction (within 1 month: adjusted HR = 0.390, 95% CI = 0.284–0.535; 1 month to 1 year: adjusted HR = 0.546, 95% CI = 0.398–0.748).

**Conclusions:**

Patients with PCL tears have a significantly higher risk of meniscus tear, OA and subsequent TKR than patients without PCL tears. PCL reconstruction could decrease the cumulative incidence of these outcomes. The results suggest that PCL-injured patients should undergo reconstruction as early as possible (within one year) to reduce the risk of further degeneration.

## Introduction

In knee injuries, posterior cruciate ligament (PCL) tears are much rarer than anterior cruciate ligament tears. The previous studies have reported an incidence between 1 and 47% for PCL injuries in acute knee ligament injuries [1–6] Although many studies have focused on the anterior cruciate ligament, the natural history, treatment and outcomes of a PCL-deficient knee have not been clearly described.[7] The appropriate treatment for isolated PCL injuries remains a controversial topic in knee surgery.[7–10] Traditionally, nonoperative treatment of isolated PCL tears has been recommended, and reconstruction surgery has been reserved for cases of persistent instability or multifilament injuries.[11–13] However, another debate remains regarding the topic of subjective knee function and subsequent osteoarthritic changes in PCL-deficient knees. [14–17]

An epidemiological study found that a history of knee injury is correlated with an increased risk of osteoarthritis (OA).[18] Another long-term follow-up study reported that patients with knee injury had at least a 5-fold increased risk of developing knee OA.[19] Previous studies have reported that 50% of patients underwent surgery for isolated ACL injuries, and 26% underwent surgery for isolated PCL injuries.[20–22] Patients with isolated PCL tears may have few functional problems; however, in one study, 26% of patients reported residual instability.[17] Many studies of PCL insufficiency focus on the problem of functional instability, and few emphasize the potential for increasing degenerative arthritis. However, instability may not be the major problem after PCL injury. Chronic pain, activity discomfort, and joint effusion may begin after early articular cartilage degeneration.

One study suggests that PCL-deficient knees are at risk of subsequent meniscal injury or arthritis.[23] In a biomechanical cadaveric study, increased strain on both the medial and lateral menisci in PCL-deficient knees was reported.[24] Another retrospective review of PCL-deficient patients 13 years after injury reported a 21% rate of subsequent meniscal tears; as the time since the injury increased, increased articular degeneration was observed on radiographs.[25]

In recent years, PCL reconstruction has become an increasingly common choice for treatment to reduce posterior laxity. However, it is still unclear whether PCL reconstruction will improve subjective symptoms or prevent OA compared with nonoperative treatment, and previous studies have had limitations such as small sample sizes or short follow-up times.

This nationwide cohort study with a long follow-up used data from the National Health Insurance Research Database (NHIRD) in Taiwan to estimate the risk of poor prognoses (including subsequent meniscus tear, OA and total knee replacement (TKR) surgery) in the knee after PCL injury for patients treated surgically compared with those treated non-surgically.

## Methods

### Data sources

Data from the NHIRD were used to investigate the risk of progressive meniscus tear, OA and TKR surgery in patients with PCL injury with or without PCL reconstruction over a 15-year period. The data were drawn from the Longitudinal Health Insurance Database in Taiwan (2000–2015), which is a valid representative sample of the country’s total population of 23,000,000. The Taiwan National Health Insurance Program has been in place since 1995, and as of June 2009, it included contracts with 97% of medical providers and approximately 23 million individuals, more than 99% of the entire population in Taiwan.[26] The NHIRD contains all hospital outpatient and inpatient medical payment records and uses International Classification of Diseases, 9th Revision, Clinical Modification (ICD-9-CM) codes to record diagnoses. Several studies have demonstrated the accuracy and validity of the diagnoses in the NHIRD.[27–30] This study used Longitudinal Health Insurance Database 2000 (LHID2000) for further analysis. LHID2000 is a subset database of the NHIRD and comprising random cases of two million people included in the NHIRD data from 2000. The sex distribution between the LHID2000 and NHIRD showed no significant difference (χ2 = 1.74, df = 1, p-value = 0.187). The Institutional Review Board of Tri-service General Hospital approved this study (TSGHIRB No. 2-104-05-126) and the committee waived the need for written informed consent.

### Study design and population

**Fig 1:**
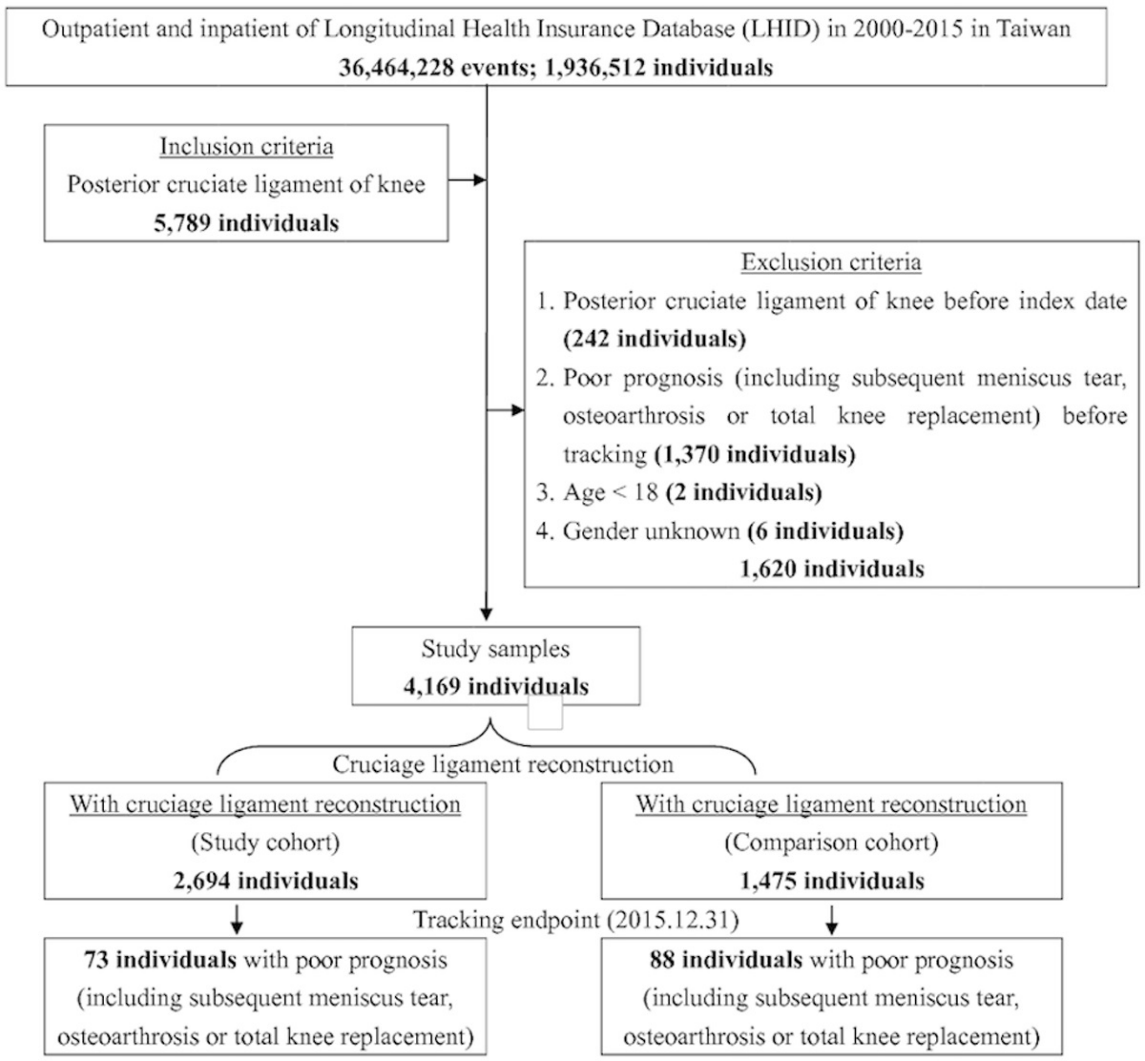
The flowchart of study sample selection from National Health Insurance Research Database in Taiwan.

This cohort study investigated patients with PCL injury who underwent PCL reconstruction surgery and a reference group of patients with PCL injury who received conservative treatment during the evaluation period. We evaluated the risks of meniscus tear, OA and TKR surgery in PCL injury patients who underwent PCL reconstruction surgery and those who did not. We used the ICD-9-CM codes to identify diagnoses in the NHIRD related to PCL, meniscus tear, OA and total knee replacement. Patients with PCL injury (defined by ICD-9-CM code 717.84) reported before December 31, 2015, were included in the analyses. There was a total of 5,789 patients with PCL injury in the database. The first date of PCL injury diagnosis was defined as the date of onset. All the subjects were followed up from the onset of PCL injury until the onset of meniscus tear (ICD-9-CM 710.0–710.5, 836.0–836.2), OA (ICD-9-CM codes 715.16, 715.26, 715.36, or 715.96), total knee replacement (ICD-9-CM OP81.54) or the end of 2015. The exclusion criteria were unknown sex (6 patients); age less than 18 years at the onset of PCL injury (2 patients); date of meniscus tear, OA and TKR onset prior to PCL injury (1370 patients); and reconstruction date before the date of the PCL injury (242 patients). Finally, a total of 4,169 patients with PCL injury were included in the study analysis. A flow diagram of the study cohort selection and study design is provided in Fig 1.

### Statistical analysis

All analyses were performed using SPSS software version 22 (SPSS, Inc., Chicago, Illinois, USA). χ2 and t tests were used to evaluate the distributions of categorical and continuous variables, respectively. Multivariable Cox proportional hazards regression models was used to determine the risk of meniscus tear, OA and total knee replacement; the results are presented as HRs with 95% CIs. The differences in the risk of subsequent meniscus tear, OA and TKR between the two groups (with or without PCL reconstruction) was estimated using the Kaplan-Meier method with the log-rank test. A 2-tailed P value < 0.05 was considered significant.

## Results

### Prevalence of meniscus tear, osteoarthritis and total knee replacement in patients with and without PCL injury

According to the assessed data from January 1, 2000, to December 31, 2015, a total of 5,789 patients were diagnosed with PCL injury, and 4,169 patients fulfilled the eligibility criteria. As Table 1 shows, we identified 4,169 patients with PCL injury and 16,676 patients for the non-PCL injury comparison cohort. No significant differences were observed in sex and age at baseline. At the end of follow-up (Table 1), 47 (1.13%, 47/4,169) patients in the PCL injury group had developed a meniscus tear, compared with 37 (0.22%, 37/16,676) in the control group; 113 (2.71%, 113/4,169) patients in the PCL injury group developed knee osteoarthritis, compared with 317 (1.90%, 317/16,676) in the control group; and 38 (0.91%, 38/4,169) patients in the PCL injury group received TKR, compared with 103 (0.62%, 103/16,676) in the control group. All these results showed statistically significant differences (P *<* 0.05).

**Table 1:** Characteristics of patients with or without PCL injury.

Posterior cruciate ligament	Total		With		Without		P
Variables	n	%	n	%	n	%	
**Total**	20,845		4,169	20.00	16,676	80.00	
**Sex**							0.999
Male	14,765	70.83	2,953	70.83	11,812	70.83	
Female	6,080	29.17	1,216	29.17	4,864	29.17	
**Age (years)**	32.54±17.94		32.15±12.65		32.64±19.03		0.115
**Age group (years)**							0.999
18–24	8,815	42.29	1,763	42.29	7,052	42.29	
25–49	9,585	45.98	1,917	45.98	7,668	45.98	
≥50	2,445	11.73	489	11.73	1,956	11.73	
**Poor prognosis**^**a**^							<0.001
Without	20,332	97.54	4,008	96.14	16,324	97.89	
With	513	2.46	161	3.86	352	2.11	
**Meniscus tear**							<0.001
Without	20,761	99.60	4,122	98.87	16,639	99.78	
With	84	0.40	47	1.13	37	0.22	
**Osteoarthritis**							0.001
Without	20,415	97.94	4,056	97.29	16,359	98.10	
With	430	2.06	113	2.71	317	1.90	
**Total knee replacement**							0.041
Without	20,704	99.32	4,131	99.09	16,573	99.38	
With	141	0.68	38	0.91	103	0.62	

a. Poor prognosis include meniscus tear, osteoarthritis and total knee replacement.

### Characteristics of the prevalence, covariates, and comorbidities at the end of follow-up for patients with PCLR compared with those without PCLR

Of the patients with PCL injuries, 2,694 underwent PCL reconstruction surgery (the PCLR group); and 1,475 patients received conservative treatment and served as the control group (Fig 1). In our study, no significant differences in sex were observed between the group that underwent surgery and the controls at baseline. At the end of follow-up (Table 2), 11 (0.41%, 11/2,694) patients in the PCLR group developed meniscus tear, compared with 36 (2.44%, 36/1,475) in the control group; 62 (2.30%, 62/2,694) patients in the PCLR group developed knee OA, compared with 51 (3.46%, 51/1,475) in the control group; and 13 (0.48%, 13/2,694) patients in the PCLR group had TKR, compared with 25 (1.69%, 25/1,475) in the control group. All these results showed statistically significant differences (P *<* 0.05). There were no significant differences between the PCLR and control groups in sex, covariates and comorbidities, but there was a statistically significant difference in age groups (P *<* 0.001).

**Table 2:** Characteristics of PCL-injured patients with and without reconstruction.

Cruciage ligament reconstruction	Total		With		Without		*P*
Variables	n	%	n	%	n	%	
**Total**	4,169		2,694	64.62	1,475	35.38	
**Sex**							0.593
Male	2,953	70.83	1,916	71.12	1,037	70.31	
Female	1,216	29.17	778	28.88	438	29.69	
**Age (years)**	32.15±12.65		31.26±11.90		33.79±13.78		<0.001
**Age group (years)**							<0.001
18–24	1,763	42.29	1,159	43.02	604	40.95	
25–49	1,917	45.98	1,275	47.33	642	43.53	
≥50	489	11.73	260	9.65	229	15.53	
**Poor prognosis**^**a**^							<0.001
Without	4,008	96.14	2,621	97.29	1,387	94.03	
With	161	3.86	73	2.71	88	5.97	
**Meniscus tear**							<0.001
Without	4,122	98.87	2,683	99.59	1,439	97.56	
With	47	1.13	11	0.41	36	2.44	
**Osteoarthritis**							0.036
Without	4,056	97.29	2,632	97.7	1,424	96.54	
With	113	2.71	62	2.3	51	3.46	
**Total knee replacement**							<0.001
Without	4,131	99.09	2,681	99.52	1,450	98.31	
With	38	0.91	13	0.48	25	1.69	

a. Poor prognosis include meniscus tear, osteoarthritis and total knee replacement.

### Kaplan-Meier model for cumulative risk of meniscus tear, osteoarthritis and total knee replacement in the groups with and without PCLR

**Fig 2:**
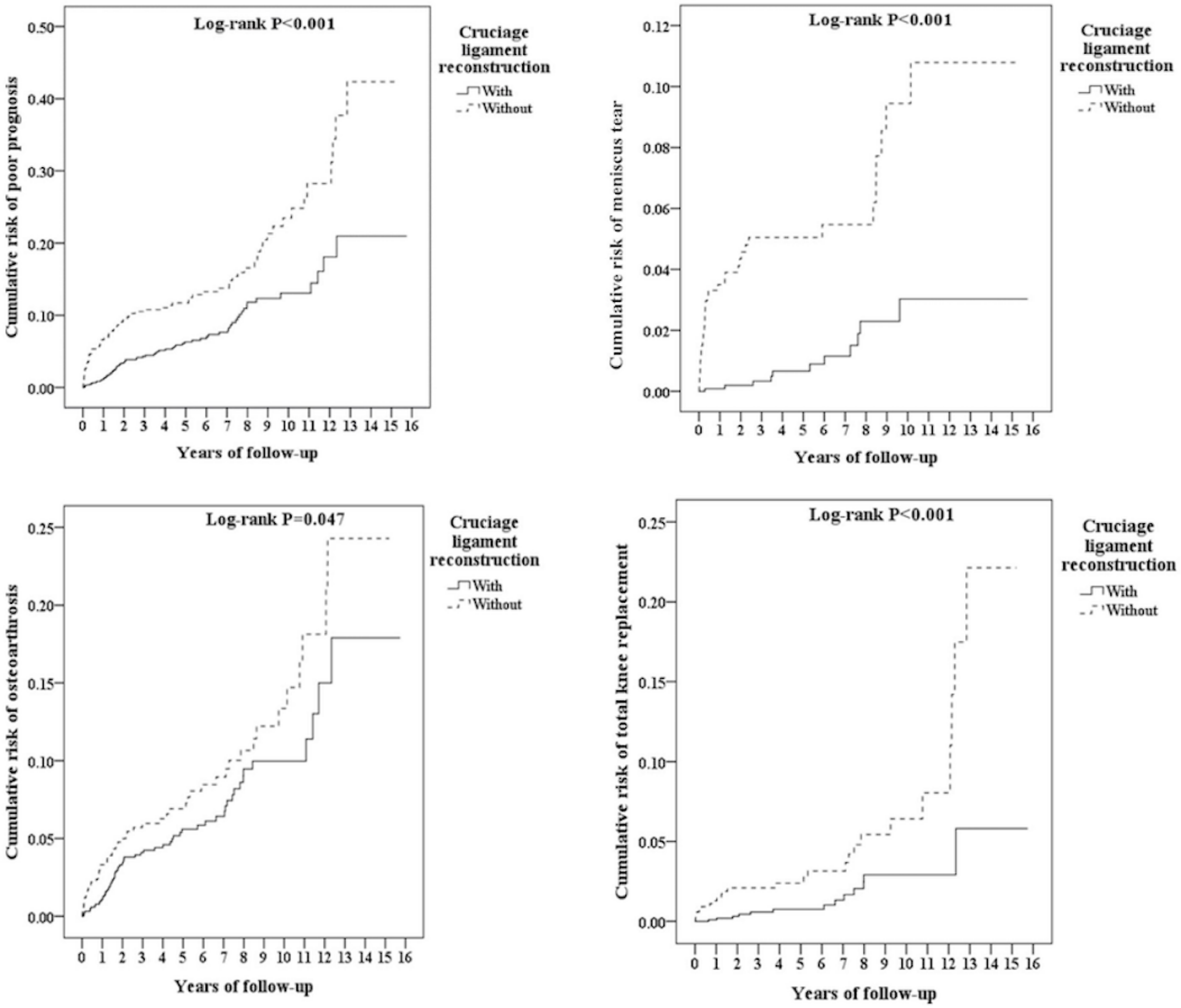
Kaplan-Meier for cumulative risk of poor prognosis among posterior cruciate ligament of knee aged 18 and over stratified by cruciate ligament reconstruction with log-rank test. Left-top: Total poor prognosis. Right-top: Meniscus tear. Left-button: Osteoarthrosis. Right-button: Total knee replacement.

The cumulative incidence curve for meniscus tear, OA and TKR in PCLR group were all significantly lower than that for the control group over the 15-year follow-up period, after adjustment for age, sex, and comorbidities (Fig 2, log-rank test; P *<* 0.05). In the control patients, the risk of meniscus tear, OA and TKR increased progressively with the duration of follow-up and remained persistently higher than that of the PCLR group.

### HRs and incidences of poor prognoses stratified by sex, age group, and comorbidities by using Cox regression for patients with PCLR compared with controls

Cox proportional hazards regressions analysis revealed a decreased risk of poor prognoses (including meniscus tear, OA and TKR), with an adjusted HR of 0.468 (95% CI, 0.341–0.642), in the PCLR group after adjustment for sex, age, and comorbidities (Table 3). No significant difference was noted between males and females. Compared with patients who had PCL injury at younger than 25 years, those who experienced PCL injury at age 25 to 50 years or older than 50 years exhibited approximately 0.4-fold (adjusted HR = 0.452, 95% CI = 0.288–0.710) and 0.7-fold (adjusted HR = 0.788, 95% CI = 0.477–1.300) lower risks of OA, respectively. Further, we analyzed the association between late PCL reconstruction and the risk of poor prognoses (Table 4). After adjusting for covariates, the PCL-injured patients who underwent reconstruction within one year after PCL injury showed a significantly lower risk of poor prognoses than those who never received reconstruction (within 1 month: adjusted HR = 0.390, 95% CI = 0.284–0.535; 1 month to 1 year: adjusted HR = 0.546, 95% CI = 0.398–0.748).

**Table 3:** HRs for poor prognoses in PCL-injured patients with and without reconstruction by using Cox regression.

Variables	Crude HR	95% CI	95% CI	*P*	Adjusted HR	95% CI	95% CI	*P*
**Cruciage ligament reconstruction**								
Without	Reference				Reference			
With	0.513	0.247	0.771	<0.001	0.468	0.341	0.642	<0.001
**Sex**								
Male	1.098	0.897	1.779	0.725	1.075	0.769	1.504	0.672
Female	Reference				Reference			
**Age group (years)**								
18–24	Reference				Reference			
25–49	0.561	0.197	0.842	0.003	0.452	0.288	0.710	0.001
≥50	0.789	0.499	1.265	0.375	0.788	0.477	1.300	0.351
**HTN**								
Without	Reference				Reference			
With	1.505	0.884	2.972	0.343	1.402	0.798	2.463	0.240
**DM**								
Without	Reference				Reference			
With	1.142	0.897	1.887	0.297	1.254	1.091	1.707	0.141
**Depression**								
Without	Reference				Reference			
With	0.000	-	-	0.897	0.000	-	-	0.964
**Anxiety**								
Without	Reference				Reference			
With	0.000	-	-	0.972	0.000	-	-	0.961

HR = hazard ratio, CI = confidence interval, Adjusted HR: Adjusted variables listed in the table

**Table 4:** Factors of poor prognosis by using Cox regression.

Events	Poor prognosis				Meniscus tear				Osteoarthritis				Total knee replacement			
Group	Adjusted HR	95% CI	95% CI	P	Adjusted HR	95% CI	95% CI	P	Adjusted HR	95% CI	95% CI	P	Adjusted HR	95% CI	95% CI	P
Without cruciage ligament reconstruction	Reference				Reference				Reference				Reference			
With cruciage ligament reconstruction	0.468	0.341	0.642	<0.001	0.172	0.126	0.236	0.012	0.686	0.500	0.941	0.001	0.293	0.214	0.402	<0.001
Without cruciage ligament reconstruction	Reference				Reference				Reference				Reference			
Time to cruciage ligament reconstruction: < 1month	0.390	0.284	0.535	<0.001	0.000	1.256×10^−18^	798.675	0.798	0.661	0.482	0.907	0.011	0.319	0.232	0.437	0.001
Time to cruciage ligament reconstruction: >1 month, <1 year	0.546	0.398	0.748	<0.001	0.000	2.421×10^−17^	370.244	0.598	0.941	0.686	1.291	0.217	0.000	3.786×10^−20^	1,316.752	0.768
Time to cruciage ligament reconstruction: ≥1 year, <3 years	2.404	1.752	3.298	0.002	3.526	2.569	4.837	0.006	2.489	1.813	3.414	0.001	2.115	1.541	2.902	0.001
Time to cruciage ligament reconstruction: ≥ 3 years	2.615	1.905	3.587	0.011	6.392	4.657	8.768	0.007	0.000	9.856×10^−19^	1,006.243	0.682	0.000	5.362×10^−20^	2,047.866	0.844

HR = hazard ratio, CI = confidence interval, Adjusted HR: Adjusted for the variables listed in Table 3.

## Discussion

To date, there has been a paucity of information on the long-term outcomes of PCL-deficient patients. [4, 11] The present study provides an initial report of the incidence of meniscal tears, arthritis and the need for TKR in a population-based sample of patients with PCL tears compared to matched subjects without PCL tears. In this cohort, PCL injuries were more common in males than females, which is comparable to previous reports.[4, 8, 31] The rates of secondary meniscal tears, arthritis and the need for TKR were all significantly higher than those of the matched subjects without PCL tears.

The progression of degenerative articular cartilage changes in patients with PCL-deficient knees is still a topic of debate in current studies. Previous studies have indicated that the PCL-deficient knee could be well tolerated and preferred a conservative treatment in most cases.[1, 3, 15, 16] Fowler et al.[16] reviewed 13 PCL-injured patients with a mean 2.6 years follow-up and found acceptable functional stability in these patients; they indicated that non-operative treatment may be a viable alternative. Shelbourne et al.[15] followed 215 PCL-deficient patients with a mean time of 7.8 years after injury and found that their subjective scores were less than normal, but subjective knee function did not worsen with time. Patel et al.[1] followed 57 patients with PCL injuries with an average follow–up of 6.9 years and noted the grade of PCL laxity had no significant correlation with the Lysholm-II knee scores; however, radiographs revealed mild degenerative changes in the medial compartment (grade I) in 7 and moderate degenerative changes (grade II) in 3.

Although previous results have shown that patients may tolerate a PCL-deficient knee with relatively mild symptoms, many studies have revealed altered loads and kinematics in the PCL-deficient knee. Logan et al.[32] revealed alterations in the kinematics of the medial compartment of the knee, resulting in anterior subluxation of the medial femoral condyle with an increased incidence of OA in the medial compartment. Hewett et al.[33] observed sagittal translation (compared with the non-involved knee) at both the medial and lateral tibial plateaus. Skyhar et al.[34] concluded that higher loads acted on the patellofemoral and medial compartment after sectioning of the PCL. Van de Velde et al.[35] used MRI and fluoroscopy to evaluate changes in tibiofemoral mechanics and found a more anterior and medial location of peak cartilage deformation on the tibial plateau. These facts show that increased forces act on the cartilage of the medial compartment because contact areas change; these changes may lead to the development of medial articular lesions in the PCL-deficient knee.

The natural course of the PCL-deficient knee, particularly the progression of degenerative articular cartilage changes, is still a focus of current debate. Parolie et al.[3] suggested that isolated PCL injuries may not lead to degenerative cartilage changes. However, more recent studies have found that PCL tears can cause meniscus and cartilage damage with a greater risk of the onset of OA.[13, 14, 17, 36–39] Geissler et al.[40] studied intraarticular abnormalities in 88 PCL-deficient knees and found articular chondral defects at the medial femoral condyle in 49% of chronic PCL-deficient knees with 65.5 months follow-up. Strobel et al.[41] evaluated 181 patients with non-surgically treated PCL injuries and found that 77.8% had degenerative cartilage lesions in the medial femoral condyle and 46.7% had degenerative lesions in the patella. Shino et al.[38] reported that 4 out of 22 PCL-deficient knees presented articular cartilage lesions at the medial femoral condyle. Hamada et al.[39] found meniscal tears in 17 of 61 cases (28%) and cartilage lesions in 32 of 61 knees (52%) after PCL injury. These findings clearly show that PCL insufficiency increases the likelihood of cartilage degeneration over time.

Our reports also found that PCL deficiency leads to the progression of meniscus tears and osteoarthritic changes after injury. These results raise concerns regarding the need to restore stability with PCL reconstruction to avoid progressive degeneration. The aim of ligamentous reconstructions is to restore the biomechanics and normal loads of the knee to regain stability and prevent further meniscus or cartilage damage. Previous reports have shown that PCL reconstruction results in improved subjective knee scores. Jackson et al[42] studied the long-term outcomes of 26 patients who underwent PCL reconstruction using hamstring tendon autografts; they reported that 24 patients rated their knees as near-normal, while 25 reported no pain or giving way of the knee with moderate to strenuous activity at a minimum of 10 years post reconstruction. Ahn et al[43] evaluated 61 patients who had undergone PCL reconstruction for a mean period of 40.8 months and reported that International Knee Documentation Committee subjective evaluations rated all patients as normal or nearly normal, and the objective evaluation revealed normal or nearly normal function in 59 patients and abnormal function in 2 patients. Sekiya et al[44] evaluated twenty-one PCL reconstruction patients with 5.9 years following up and found that more than 50% had normal/nearly normal functional scores and activity levels based on the International Knee Documentation Committee (IKDC) assessment. These findings show that patients who undergo PCL reconstruction experience a satisfactory return to daily activities.

There were few studies evaluated the association of with /without PCL reconstruction and the progression of degenerative changes.[37, 45] Hermans et al.[37] evaluated 25 patients who underwent PCL reconstruction with a mean follow-up time of 9.1 years and found no differences between the chondrosis and non-chondrosis groups on the initial radiographic findings. Only 18% of the patients in the non-chondrosis groups developed mild radiographic abnormal findings; however 92% of the patients in the chondrosis group progressed to mild to moderate abnormal radiographic findings. This study concluded that in the absence of chondrosis, the onset to radiographically detectable osteoarthritis was prevented in the majority of patients with PCL reconstructions at long-term follow-up. In our study, the rates of secondary meniscal tears, arthritis and the need for TKR were all significantly lower than those of the matched subjects without PCL reconstruction (Fig 2), which suggest that PCL reconstruction has the protective effect in preventing further degeneration of knee joint in patients with PCL tear.

A previous study also noted that extending the non-operative treatment period for more than 1 year in patients with posterior instability could decrease the long-term functional results of PCL reconstructions.[37] The study showed that 75% patients in the non-operative group tended to have more cartilage damage compared with the patients in the PCLR group treated within one year after PCL tear (41%). Delayed reconstruction resulted in worse functional scores and final radiographic findings. The final results suggest the benefit of not delaying surgery for more than 1 year after the injury. In the present study, the analyses showed that PCL-injured patients with late reconstruction had a higher risk of OA, and patients who underwent early PCL reconstruction (within 1 year after PCL injury) had a significant decline in OA risk. The cumulative incidence of TKR was lower in the patients who underwent PCL reconstruction. As the present study showed that PCL reconstruction also protected patients from osteoarthritic change, it is reasonable to propose that PCL reconstruction might delay the timing of TKR.

The current study has several limitations. The data from the NHIRD were obtained from a claims-based database; thus, the precise diagnoses and severity of PCL injury, meniscus tear or osteoarthritic change could not be determined. In addition, the data could not be used to determine the type of surgical methods and grafts used for PCL reconstruction. Meniscus tear and TKR were only used as the following-up outcome to reveal the severity of OA. Some potential confounding variables (such as trauma history and BMI) were also difficult to be accessed, because the NHIRD lacks these data. The following period from 20000 to 2015 is relative not long enough to evaluate really long-term outcome, and longer following-up period is still needed in the future study. The strengths of the present study include that the national insurance claims database covered all medical clinics and hospitals in our country, and a random sample of a consistent percentage could be obtained from all hospitals. The other advantages of the present study include the large sample size and the use of precise ICD-9-CM codes for the diagnosis of disease and following-up treatment.

## Conclusion

In the present study, we found that patients with PCL tears have a significantly higher risk of meniscus tear, arthritis and TKR than matched peers without PCL tears. Patients who had undergone PCL reconstruction had a decreased incidence of meniscus tear, OA and TKR surgery compared with patients who were treated conservatively. We further observed that patients who underwent reconstruction within 1 year after PCL injury experienced a significant protective effect against these poor prognoses. The results of this study can provide a baseline of expectations for the future development of meniscal injury and arthritis following PCL injury with and without PCL reconstruction.

## References

[1] Patel DV, Allen AA, Warren RF, Wickiewicz TL, Simonian PT. The nonoperative treatment of acute, isolated (partial or complete) posterior cruciate ligament-deficient knees: an intermediate-term follow-up study. HSS journal: the musculoskeletal journal of Hospital for Special Surgery. 2007;3(2):137–46. Epub 2008/08/30. 10.1007/s11420-007-9058-z ; PubMed Central PMCID: PMCPMC2504260. 18751784 PMC2504260

[2] Hughston JC, Degenhardt TC. Reconstruction of the posterior cruciate ligament. Clinical orthopaedics and related research. 1982;(164):59–77. Epub 1982/04/01. . 7073822

[3] Parolie JM, Bergfeld JA. Long-term results of nonoperative treatment of isolated posterior cruciate ligament injuries in the athlete. The American journal of sports medicine. 1986;14(1):35–8. Epub 1986/01/01. 10.1177/036354658601400107 . 3752344

[4] Schulz MS, Russe K, Weiler A, Eichhorn HJ, Strobel MJ. Epidemiology of posterior cruciate ligament injuries. Archives of orthopaedic and trauma surgery. 2003;123(4):186–91. Epub 2003/05/08. 10.1007/s00402-002-0471-y . 12734718

[5] Fanelli GC, Giannotti BF, Edson CJ. The posterior cruciate ligament arthroscopic evaluation and treatment. Arthroscopy: the journal of arthroscopic & related surgery: official publication of the Arthroscopy Association of North America and the International Arthroscopy Association. 1994;10(6):673–88. Epub 1994/12/01. . 7880360 10.1016/s0749-8063(05)80067-2

[6] Majewski M, Susanne H, Klaus S. Epidemiology of athletic knee injuries: A 10-year study. The Knee. 2006;13(3):184–8. Epub 2006/04/11. 10.1016/j.knee.2006.01.005 . 16603363

[7] Watsend AM, Osestad TM, Jakobsen RB, Engebretsen L. Clinical studies on posterior cruciate ligament tears have weak design. Knee surgery, sports traumatology, arthroscopy: official journal of the ESSKA. 2009;17(2):140–9. Epub 2008/10/18. 10.1007/s00167-008-0632-9 . 18925355

[8] LaPrade CM, Civitarese DM, Rasmussen MT, LaPrade RF. Emerging Updates on the Posterior Cruciate Ligament: A Review of the Current Literature. The American journal of sports medicine. 2015;43(12):3077–92. Epub 2015/03/18. 10.1177/0363546515572770 . 25776184

[9] Shelbourne KD, Clark M, Gray T. Minimum 10-year follow-up of patients after an acute, isolated posterior cruciate ligament injury treated nonoperatively. The American journal of sports medicine. 2013;41(7):1526–33. Epub 2013/05/09. 10.1177/0363546513486771 . 23652263

[10] Hammoud S, Reinhardt KR, Marx RG. Outcomes of posterior cruciate ligament treatment: a review of the evidence. Sports medicine and arthroscopy review. 2010;18(4):280–91. Epub 2010/11/17. 10.1097/JSA.0b013e3181eaf8b4 . 21079509

[11] Cosgarea AJ, Jay PR. Posterior cruciate ligament injuries: evaluation and management. The Journal of the American Academy of Orthopaedic Surgeons. 2001;9(5):297–307. Epub 2001/09/29. . 11575909 10.5435/00124635-200109000-00003

[12] Bedi A, Musahl V, Cowan JB. Management of Posterior Cruciate Ligament Injuries: An Evidence-Based Review The Journal of the American Academy of Orthopaedic Surgeons. 2016;24(5):277–89. Epub 2016/04/21. 10.5435/JAAOS-D-14-00326 . 27097125

[13] Wind WM Jr., Bergfeld JA, Parker RD. Evaluation and treatment of posterior cruciate ligament injuries: revisited. The American journal of sports medicine. 2004;32(7):1765–75. Epub 2004/10/21. 10.1177/0363546504270481 . 15494347

[14] Keller PM, Shelbourne KD, McCarroll JR, Rettig AC. Nonoperatively treated isolated posterior cruciate ligament injuries. The American journal of sports medicine. 1993;21(1):132–6. Epub 1993/01/01. 10.1177/036354659302100122 . 8427355

[15] Shelbourne KD, Muthukaruppan Y. Subjective results of nonoperatively treated, acute, isolated posterior cruciate ligament injuries. Arthroscopy: the journal of arthroscopic & related surgery: official publication of the Arthroscopy Association of North America and the International Arthroscopy Association. 2005;21(4):457–61. Epub 2005/04/01. 10.1016/j.arthro.2004.11.013 . 15800527

[16] Fowler PJ, Messieh SS. Isolated posterior cruciate ligament injuries in athletes. The American journal of sports medicine. 1987;15(6):553–7. Epub 1987/11/01. 10.1177/036354658701500606 . 3425783

[17] Shelbourne KD, Davis TJ, Patel DV. The natural history of acute, isolated, nonoperatively treated posterior cruciate ligament injuries. A prospective study. The American journal of sports medicine. 1999;27(3):276–83. Epub 1999/06/03. 10.1177/03635465990270030201 . 10352760

[18] Davis MA, Ettinger WH, Neuhaus JM, Cho SA, Hauck WW. The association of knee injury and obesity with unilateral and bilateral osteoarthritis of the knee. American journal of epidemiology. 1989;130(2):278–88. Epub 1989/08/01. . 2750727 10.1093/oxfordjournals.aje.a115334

[19] Gelber AC, Hochberg MC, Mead LA, Wang NY, Wigley FM, Klag MJ. Joint injury in young adults and risk for subsequent knee and hip osteoarthritis. Annals of internal medicine. 2000;133(5):321–8. Epub 2000/09/09. . 10979876 10.7326/0003-4819-133-5-200009050-00007

[20] Aroen A, Sivertsen EA, Owesen C, Engebretsen L, Granan LP. An isolated rupture of the posterior cruciate ligament results in reduced preoperative knee function in comparison with an anterior cruciate ligament injury. Knee surgery, sports traumatology, arthroscopy: official journal of the ESSKA. 2013;21(5):1017–22. Epub 2012/07/18. 10.1007/s00167-012-2132-1 . 22801932

[21] Jung YB, Tae SK, Lee YS, Jung HJ, Nam CH, Park SJ. Active non-operative treatment of acute isolated posterior cruciate ligament injury with cylinder cast immobilization. Knee surgery, sports traumatology, arthroscopy: official journal of the ESSKA. 2008;16(8):729–33. Epub 2008/04/18. 10.1007/s00167-008-0531-0 . 18418575

[22] Granan LP, Engebretsen L, Bahr R. [Surgery for anterior cruciate ligament injuries in Norway]. Tidsskrift for den Norske laegeforening: tidsskrift for praktisk medicin, ny raekke. 2004;124(7):928–30. Epub 2004/04/03. . 15060639

[23] Okazaki K, Takayama Y, Osaki K, Matsuo Y, Mizu-Uchi H, Hamai S, et al Subclinical cartilage degeneration in young athletes with posterior cruciate ligament injuries detected with T1rho magnetic resonance imaging mapping. Knee surgery, sports traumatology, arthroscopy: official journal of the ESSKA. 2015;23(10):3094–100. Epub 2014/12/08. 10.1007/s00167-014-3469-4 . 25481808

[24] Pearsall AWt Hollis JM. The effect of posterior cruciate ligament injury and reconstruction on meniscal strain. The American journal of sports medicine. 2004;32(7):1675–80. Epub 2004/10/21. 10.1177/0363546504265937 . 15494332

[25] Boynton MD, Tietjens BR. Long-term followup of the untreated isolated posterior cruciate ligament-deficient knee. The American journal of sports medicine. 1996;24(3):306–10. Epub 1996/05/01. 10.1177/036354659602400310 . 8734880

[26] Ho Chan WS. Taiwan's healthcare report 2010. The EPMA journal. 2010;1(4):563–85. Epub 2010/12/01. 10.1007/s13167-010-0056-8 ; PubMed Central PMCID: PMCPMC3405348. 23199110 PMC3405348

[27] Cheng CL, Kao YH, Lin SJ, Lee CH, Lai ML. Validation of the National Health Insurance Research Database with ischemic stroke cases in Taiwan. Pharmacoepidemiology and drug safety. 2011;20(3):236–42. Epub 2011/02/26. 10.1002/pds.2087 . 21351304

[28] Hsieh CY, Chen CH, Li CY, Lai ML. Validating the diagnosis of acute ischemic stroke in a National Health Insurance claims database. Journal of the Formosan Medical Association = Taiwan yi zhi. 2015;114(3):254–9. Epub 2013/10/22. 10.1016/j.jfma.2013.09.009 . 24140108

[29] Huang YJ, Huang TW, Lin FH, Chung CH, Tsao CH, Chien WC. Radiation Therapy for Invasive Breast Cancer Increases the Risk of Second Primary Lung Cancer: A Nationwide Population-Based Cohort Analysis. Journal of thoracic oncology: official publication of the International Association for the Study of Lung Cancer. 2017;12(5):782–90. Epub 2017/02/20. 10.1016/j.jtho.2017.01.021 . 28214559

[30] Lin CL, Liu TC, Lin FH, Chung CH, Chien WC. Association between sleep disorders and hypertension in Taiwan: a nationwide population-based retrospective cohort study. Journal of human hypertension. 2017;31(3):220–4. Epub 2016/08/12. 10.1038/jhh.2016.55 . 27511477

[31] Sanders TL, Pareek A, Barrett IJ, Kremers HM, Bryan AJ, Stuart MJ, et al Incidence and long-term follow-up of isolated posterior cruciate ligament tears. Knee surgery, sports traumatology, arthroscopy: official journal of the ESSKA. 2016 Epub 2016/02/29. 10.1007/s00167-016-4052-y . 26922055

[32] Logan M, Williams A, Lavelle J, Gedroyc W, Freeman M. The effect of posterior cruciate ligament deficiency on knee kinematics. The American journal of sports medicine. 2004;32(8):1915–22. Epub 2004/12/02. . 15572321 10.1177/0363546504265005

[33] Hewett TE, Noyes FR, Lee MD. Diagnosis of complete and partial posterior cruciate ligament ruptures. Stress radiography compared with KT-1000 arthrometer and posterior drawer testing. The American journal of sports medicine. 1997;25(5):648–55. Epub 1997/09/26. 10.1177/036354659702500510 . 9302470

[34] Skyhar MJ, Warren RF, Ortiz GJ, Schwartz E, Otis JC. The effects of sectioning of the posterior cruciate ligament and the posterolateral complex on the articular contact pressures within the knee. The Journal of bone and joint surgery American volume. 1993;75(5):694–9. Epub 1993/05/01. . 8501084 10.2106/00004623-199305000-00008

[35] Van de Velde SK, Bingham JT, Gill TJ, Li G. Analysis of tibiofemoral cartilage deformation in the posterior cruciate ligament-deficient knee. The Journal of bone and joint surgery American volume. 2009;91(1):167–75. Epub 2009/01/06. 10.2106/JBJS.H.00177 ; PubMed Central PMCID: PMCPMC2663325. 19122092 PMC2663325

[36] Christel P. Basic principles for surgical reconstruction of the PCL in chronic posterior knee instability. Knee surgery, sports traumatology, arthroscopy: official journal of the ESSKA. 2003;11(5):289–96. Epub 2003/09/19. 10.1007/s00167-003-0407-2 . 13680106

[37] Hermans S, Corten K, Bellemans J. Long-term results of isolated anterolateral bundle reconstructions of the posterior cruciate ligament: a 6- to 12-year follow-up study. The American journal of sports medicine. 2009;37(8):1499–507. Epub 2009/05/20. 10.1177/0363546509333479 . 19451096

[38] Shino K, Horibe S, Nakata K, Maeda A, Hamada M, Nakamura N. Conservative treatment of isolated injuries to the posterior cruciate ligament in athletes. The Journal of bone and joint surgery British volume. 1995;77(6):895–900. Epub 1995/11/01. . 7593102

[39] Hamada M, Shino K, Mitsuoka T, Toritsuka Y, Natsu-Ume T, Horibe S. Chondral injury associated with acute isolated posterior cruciate ligament injury. Arthroscopy: the journal of arthroscopic & related surgery: official publication of the Arthroscopy Association of North America and the International Arthroscopy Association. 2000;16(1):59–63. Epub 2000/01/08. . 10627346 10.1016/s0749-8063(00)90128-2

[40] Geissler WB, Whipple TL. Intraarticular abnormalities in association with posterior cruciate ligament injuries. The American journal of sports medicine. 1993;21(6):846–9. Epub 1993/11/01. 10.1177/036354659302100615 . 8291637

[41] Strobel MJ, Weiler A, Schulz MS, Russe K, Eichhorn HJ. Arthroscopic evaluation of articular cartilage lesions in posterior-cruciate-ligament-deficient knees. Arthroscopy: the journal of arthroscopic & related surgery: official publication of the Arthroscopy Association of North America and the International Arthroscopy Association. 2003;19(3):262–8. Epub 2003/03/11. 10.1053/jars.2003.50037 . 12627150

[42] Jackson WF, van der Tempel WM, Salmon LJ, Williams HA, Pinczewski LA. Endoscopically-assisted single-bundle posterior cruciate ligament reconstruction: results at minimum ten-year follow-up. The Journal of bone and joint surgery British volume. 2008;90(10):1328–33. Epub 2008/10/02. 10.1302/0301-620X.90B10.20517 . 18827243

[43] Ahn JH, Yang HS, Jeong WK, Koh KH. Arthroscopic transtibial posterior cruciate ligament reconstruction with preservation of posterior cruciate ligament fibers: clinical results of minimum 2-year follow-up. The American journal of sports medicine. 2006;34(2):194–204. Epub 2005/11/24. 10.1177/0363546505279915 . 16303881

[44] Sekiya JK, West RV, Ong BC, Irrgang JJ, Fu FH, Harner CD. Clincial outcomes after isolated arthroscopic single-bundle posterior cruciate ligament reconstruction. Arthroscopy: the journal of arthroscopic & related surgery: official publication of the Arthroscopy Association of North America and the International Arthroscopy Association. 2005;21(9):1042–50. Epub 2005/09/21. 10.1016/j.arthro.2005.05.023 . 16171628

[45] Voos JE, Mauro CS, Wente T, Warren RF, Wickiewicz TL. Posterior cruciate ligament: anatomy, biomechanics, and outcomes. The American journal of sports medicine. 2012;40(1):222–31. Epub 2011/08/02. 10.1177/0363546511416316 . 21803977

